# Detachment stress mediated bioenergetic switch of malignant melanoma cells into anti-Warburg phenotype

**DOI:** 10.18632/aging.204164

**Published:** 2022-07-07

**Authors:** WuChing Uen, TingTing Tseng, Ching-Po Wu, ShaoChen Lee

**Affiliations:** 1 School of Medicine, Fu Jen Catholic University, Xinzhuang, New Taipei City, Taiwan; 2 Department of Hematology and Oncology, Shin Kong Wu Ho-Su Memorial Hospital, Shih-Lin, Taipei City, Taiwan

**Keywords:** glycolysis, melanoma, anti-Warburg, detachment stress, electron transport chain

## Abstract

One of the biological features of cancer cells is their aerobic glycolysis by extensive glucose fermentation to harvest energy, so called Warburg effect. Melanoma is one of the most aggressive human cancers with poor prognosis and high mortality for its high metastatic ability. During the metastatic process, the metastatic tumor cells should survive under detachment stress. However, whether the detachment stress could affect the tumor phenotype is worthy to investigate.

We had established the cell model of human melanoma cells under detachment stress, which mimicked circulating melanoma. It had been demonstrated that the detachment stress altered melanoma cell activities, malignancy, and drug sensitivity. In this study, we found that adherent melanoma cells were more sensitive to glucose depletion. Gene expression profiling altered expressions of transporters associated with glucose metabolism. In addition, detachment stress reduced lactate secretion owing to the reduced MCT4 and GLUT1 expressions, the altered glycolytic and respiratory capacities, and the increased superoxide production. Detachment stress also increases the sensitivity of melanoma cells toward the blockade of electron transport chains. Investigation of the change in glucose metabolism of melanoma cells under detachment stress would be critical to provide a novel molecular mechanism to develop potential therapeutics.

## INTRODUCTION

Detachment of tumor cells from extracellular matrix (ECM) and survival under anchorage-independent conditions is recognized as the initial step of tumor metastasis [1]. ECM detachment of normal cells causes cell apoptosis through anoikis signaling [2, 3]. However, tumor cells would survive under detachment stress by harvesting the ability of anoikis resistance and anchorage-independent growth [4]. It was suggested that anchorage-independent growth promoted oncogenic transformation through hyperactive signaling pathways associated with epithelial-mesenchymal transition. Many studies had focused on the change of protein expression for malignant metastatic cells under anchorage-independency. However, the understanding of how these changes contributed to alternative phenotypes and altered the microenvironment specificity of tumor cells was limited.

Under the aerobic conditions, normal cells generate ATP from glucose metabolism through coupled mechanisms of glycolysis, tricarboxylic acid cycle, and oxidative phosphorylation. For glycolysis, glucose oxidation by serial enzymes produces pyruvate, which enters the tricarboxylic acid cycle in the mitochondria. These processes further produce the reduced electron carriers NADH and FADH_2_, which could be further used in oxidative phosphorylation. On the other hand, tumor cells generate ATP and biosynthetic backbone through the altered metabolism relying mostly on aerobic glycolysis in the presence of abundant oxygen support [5–9]. Otto Warburg suggested the uncoupling of glycolysis with tricarboxylic acid cycle and oxidative phosphorylation led to increased lactate production, so called the Warburg effect. However, if there is any mechanism that could actively reverse the feature of Warburg effect in cancer cells, that will be interesting and worthy to be investigated.

Previously, we had studied the roles of detachment stress in melanoma cells to affect their cell activities, tumor malignancy, heterogeneity, and drug sensitivity [10–13]. In this paper, we also investigated whether the glucose metabolism and mitochondrial respiration were different between adherent and suspended melanoma cells. The drug sensitivities toward different compounds were also changed by detachment stress.

## RESULTS

### Detachment stress affected several gene expressions associated with glucose metabolism

**Figure 1:**
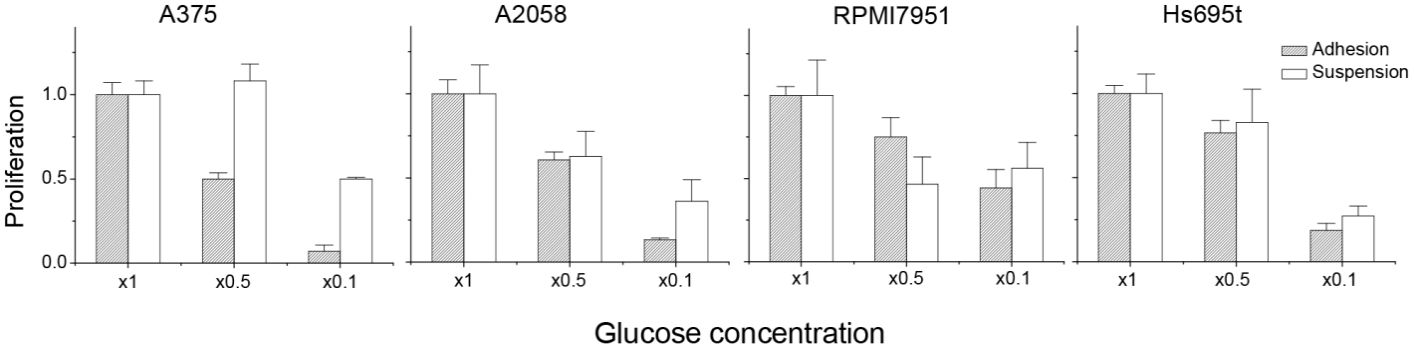
**Detachment stress contributed to the characteristics of resistance to glucose depletion.** The relative ratio of proliferation was defined as: (cell number under indicated glucose concentration) / (cell number under normal culture condition).

Previously, we had shown that reduced cell growth was observed in suspended melanoma cells compared to that of adherent melanoma cells [11]. Reduced cell growth correlated with the decrease in cell survival or delay in cell proliferation. We suspected that the suspended melanoma cells might have altered glucose metabolism compared with that in adherent melanoma cells. In general, cancer cells were more dependent on aerobic glycolysis to obtain energy, so they would be sensitive to the change in glucose concentration. To examine whether the detachment stress would alter the phenotypes of glucose-dependency, melanoma cells were cultured under different glucose concentrations. As seen in Figure 1, decreasing glucose concentration suppressed the cell proliferation of adherent melanoma cells. Reducing the glucose concentration to 1/10 of the culture condition almost blocked the cell growth of adherent cells. However, the relative ratios of cell proliferation were larger for suspended melanoma cells under lower glucose concentrations, especially in melanoma A375 cells. This indicated that suspended melanoma cells were less sensitive to the decreased glucose supplement. Either reduced glucose demand was presented in suspended melanoma cells or higher efficiency of glucose uptake might counteract the reduced glucose supply.

Thus, we examined the gene expression profiles of several glucose metabolism-related genes in four different melanoma cells under adhesion and suspension. Glucose-related gene-sets were chosen from Molecular Signatures Database [14]. First, totally 29 gene-sets associated with glucose-metabolism were selected from C5 gene ontology gene-sets using the keyword of “glucose”, which is involved in Biological Process (http://www.gsea-msigdb.org/gsea/index.jsp), as listed in Table 1. Second, all the genes in these 29 gene-sets were summarized into 1,005 different gene targets. The differential expression of these gene targets was compared in 4 different melanoma cells under adhesion and suspension (GSE42876 and GSE198509). Of all these genes, 10 genes (CAV1, IRS1, LMNA, LMNB2, PEA15, POLD1, PRKAG2, RNASEH2A, SLC29A1, and SLC2A1) were all downregulated, while 7 genes were all upregulated (AMACR, ARNT2, BBS2, FOS, FOXO1, PCSK1, and PLAG1) in suspended melanoma cells, as listed in Table 2. Most of them would involve stress-response upon cell detachment in the categories of material transport, expression/processing of gene products, and glucose homeostasis. Of note, SLC2A1 gene was known as GLUT1, which played role in direct glucose import into cells, which was responsible for glucose supply in glycolysis. Downregulation of GLUT1 in melanoma cells under detachment stress would explain their slow proliferation [10] and more resistance to low glucose supplements. Our preliminary also showed that the expression level of GLUT1 was relatively less in melanoma A375 cells than in other melanoma cells. This explained why adherent A375 cells were more sensitive to low glucose stress (Figure 1).

**Table 1:** Gene-sets associated with glucose metabolism.

**Systematic name**	**Name of gene-sets**	**# of genes**
M16733	GOBP_CELLULAR_GLUCAN_METABOLIC_PROCESS	74
M14255	GOBP_CELLULAR_GLUCOSE_HOMEOSTASIS	155
M40367	GOBP_GLUCAN_BIOSYNTHETIC_PROCESS	46
M23410	GOBP_GLUCOCORTICOID_SECRETION	11
M11917	GOBP_GLUCOSE_6_PHOSPHATE_METABOLIC_PROCESS	24
M13142	GOBP_GLUCOSE_IMPORT	79
M34267	GOBP_GLUCOSE_IMPORT_ACROSS_PLASMA_MEMBRANE	5
M40438	GOBP_GLUCOSE_IMPORT_IN_RESPONSE_TO_INSULIN_STIMULUS	5
M29050	GOBP_GLUCOSYLCERAMIDE_METABOLIC_PROCESS	7
M29123	GOBP_GLYCOLYTIC_FERMENTATION	5
M23389	GOBP_INSULIN_SECRETION_INVOLVED_IN_CELLULAR_RESPONSE_TO_GLUCOSE_STIMULUS	59
M16320	GOBP_NEGATIVE_REGULATION_OF_GLUCOSE_IMPORT	17
M22686	GOBP_NEGATIVE_REGULATION_OF_GLUCOSE_TRANSMEMBRANE_TRANSPORT	22
M11915	GOBP_PANCREAS_DEVELOPMENT	81
M23186	GOBP_POSITIVE_REGULATION_OF_GLUCOKINASE_ACTIVITY	5
M40449	GOBP_POSITIVE_REGULATION_OF_GLUCOSE_IMPORT	37
M16946	GOBP_POSITIVE_REGULATION_OF_GLUCOSE_METABOLIC_PROCESS	42
M22685	GOBP_POSITIVE_REGULATION_OF_GLUCOSE_TRANSMEMBRANE_TRANSPORT	44
M29099	GOBP_REGULATION_OF_GLUCAN_BIOSYNTHETIC_PROCESS	31
M16528	GOBP_REGULATION_OF_GLUCONEOGENESIS	51
M34192	GOBP_REGULATION_OF_GLUCOSE_IMPORT	60
M13722	GOBP_REGULATION_OF_GLUCOSE_METABOLIC_PROCESS	123
M22684	GOBP_REGULATION_OF_GLUCOSE_TRANSMEMBRANE_TRANSPORT	78
M23770	GOBP_REGULATION_OF_TRANSCRIPTION_BY_GLUCOSE	7
M15554	GOBP_RENAL_ABSORPTION	20
M29023	GOBP_UDP_GLUCOSE_METABOLIC_PROCESS	5
M34443	GOMF_D_GLUCOSE_TRANSMEMBRANE_TRANSPORTER_ACTIVITY	11
M18136	GOMF_GLUCOSIDASE_ACTIVITY	12
M26662	GOMF_PROTEIN_KINASE_B_BINDING	11

**Table 2:** Downregulated and upregulated genes in melanoma cells under detachment stress.

**Downregulated genes associated with glucose metabolism**		
**Gene symbol**	**Name**	**Molecular function**
CAV1	caveolin 1	Scaffold protein of caveolae
IRS1	insulin receptor substrate 1	Substrate of insulin receptor tyrosine kinase
LMNA	lamin A/C	Nuclear protein
LMNB2	lamin B2	Nuclear protein
PEA15	proliferation and apoptosis adaptor protein 15	Negative regulator of apoptosis
POLD1	DNA polymerase delta 1, catalytic subunit	DNA synthesis
PRKAG2	protein kinase AMP-activated non-catalytic subunit gamma 2	Glycogen storage
RNASEH2A	ribonuclease H2 subunit A	Ribonuclease component
SLC29A1	solute carrier family 29 member 1 (Augustine blood group)	Nucleoside transporter
SLC2A1	solute carrier family 2 member 1	Glucose transport
**Upregulated genes associated with glucose metabolism**		
**Gene symbol**	**Name**	**Molecular function**
AMACR	alpha-methylacyl-CoA racemase	Lipid metabolism
ARNT2	aryl hydrocarbon receptor nuclear translocator 2	Transcription factor
BBS2	Bardet-Biedl syndrome 2	Intracellular trafficking
FOS	Fos proto-oncogene, AP-1 transcription factor subunit	Transcription factor
FOXO1	forkhead box O1	Transcription factor
PCSK1	proprotein convertase subtilisin/kexin type 1	Proprotein processing
PLAG1	PLAG1 zinc finger	Transcription factor

### Detachment stress altered glucose dependency and lactate secretion through downregulation of MCT4 and GLUT1

In addition to find key proteins through target mining in glucose-related gene-sets, we also compared several well-studied monocarboxylate transporters in these 4 melanoma cells under adhesion/suspension. The relative expression level was defined as: [(intensity in suspension) - (intensity in adhesion)] / (intensity in adhesion), and listed in Table 3. The expression of GLUT1 was significantly reduced in 4 melanoma cells. MCT1 was recognized as lactate importer in endothelial cells or oxidative cancer cells, while MCT4 was highly expressed in glycolytic cancer cells [15]. MCT2 is high-affinity transporter for many monocarboxylates, such as pyruvate, lactate, ketone bodies, and short-chain fatty acids. No significant change in MCT2 expression in 4 different melanoma cells under detachment. However, MCT4 was significantly downregulated in A375 and Hs695t cells and partly reduced in suspended A2058 cells. MCT4 was a low-affinity transporter to export lactate from glycolytic cancer cells, and played role of lactate donor in metabolic symbiosis [16].

**Table 3:** Downregulation of key monocarboxylate transporters in suspended melanoma cells.

**Name**	**Gene symbol**	**A375 cells**	**A2058 cells**	**RPMI7951 cells**	**Hs695t cells**
		**Relative expression in suspended melanoma (%)**			
MCT1	(SLC16A1)	-7.6%	+0.6%	-6.7%	+14.7%
MCT2	(SLC16A7)	-11.5%	-6.4%	+15.4%	-6.5%
MCT4	(SLC16A3)	**-62.7%**	-28.6%	-2.5%	-**51.7%**
CLUT1	(SLC2A1)	**-66.0%**	**-40.7%**	**-38.4%**	**-60.0%**

**Figure 2:**
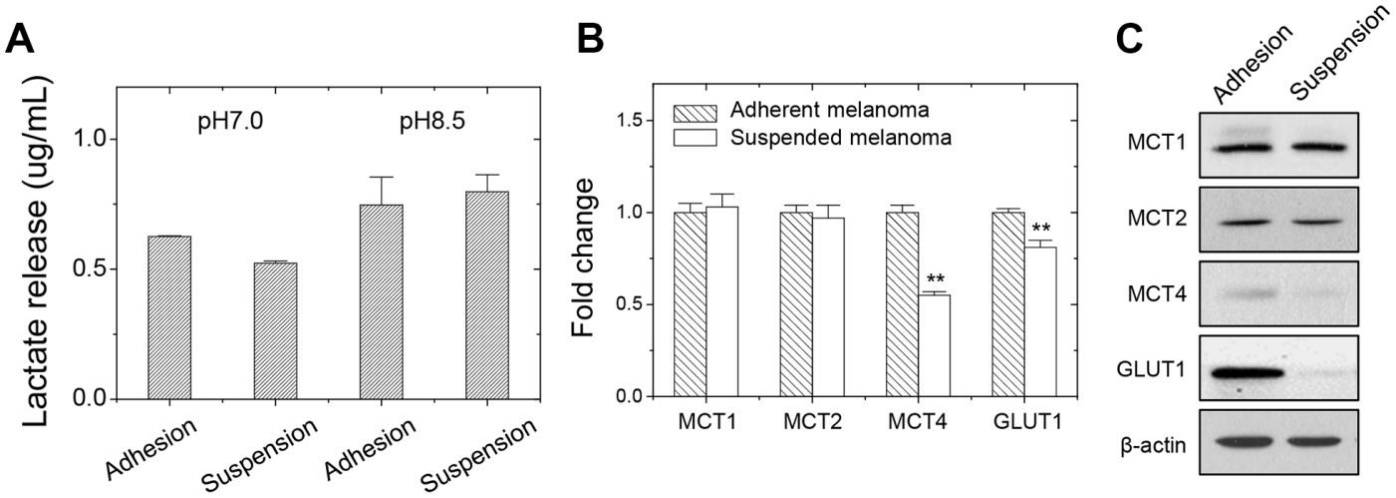
**Decreased lactate release and gene expression in suspended melanoma A375 cells.** (**A**) Reduced lactate secretion from suspended melanoma cells was seen under neutral pH condition (pH7.0). while at pH8.5, the maximum levels of lactate secretions were achieved in both adherent and suspended melanoma cells. Data were mean ±S.D. (n=3); **, *p* < 0.01. Gene expression was examined by (**B**) qPCR and (**C**) western blot. Data were mean ±S.D. (n=3); **, *p* < 0.01.

Increased glucose demand owing to increased glycolysis rate could be detected by increased lactate release. Since adherent melanoma cells were more dependent on glucose levels, we examined whether the level of lactate secretion was higher in adherent melanoma A375 cells. As seen in Figure 2A, more lactate secretion from adherent melanoma cells was observed as examining the extracellular lactate content in the conditioned medium under pH7.0. Under alkaline environment (pH8.5), the lactate release was achieved at a saturated level [17] for both adherent and suspended melanoma cells, and they showed the same levels.

We analyzed the gene expressions of carboxylate transporters. As shown in Figure 2B, qPCR analysis at adherent or suspended melanoma cells showed that MCT4 and GLUT1 expression were reduced upon detachment stress. Western blot analysis showed the MCT4 protein level, but not MCT1or MCT2 protein levels, reduced in suspended melanoma cells (Figure 2C). The reduced GLUT1 expression might correlate with the lower glucose dependency and reduced lactate secretion in suspended melanoma cells. GLUT1 protein level was also reduced in suspended melanoma cells. These suggested that less lactate secretion would derive from reduced glucose uptake and reduced lactate export. The reduced MCT4 expression in suspended melanoma cells would account for the reduced lactate secretion than that by adherent melanoma cells (Table 3 and Figure 2A).

### Detachment stress caused the metabolic switch from glycolytic pathway into aerobic pathway

Since lactate export was reduced, its effect on extracellular acidification would be suppressed. We analyzed the cellular bioenergetics of adherent and suspended melanoma cells using Seahorse Bioscience XF24 extracellular flux analyzer.

**Figure 3:**
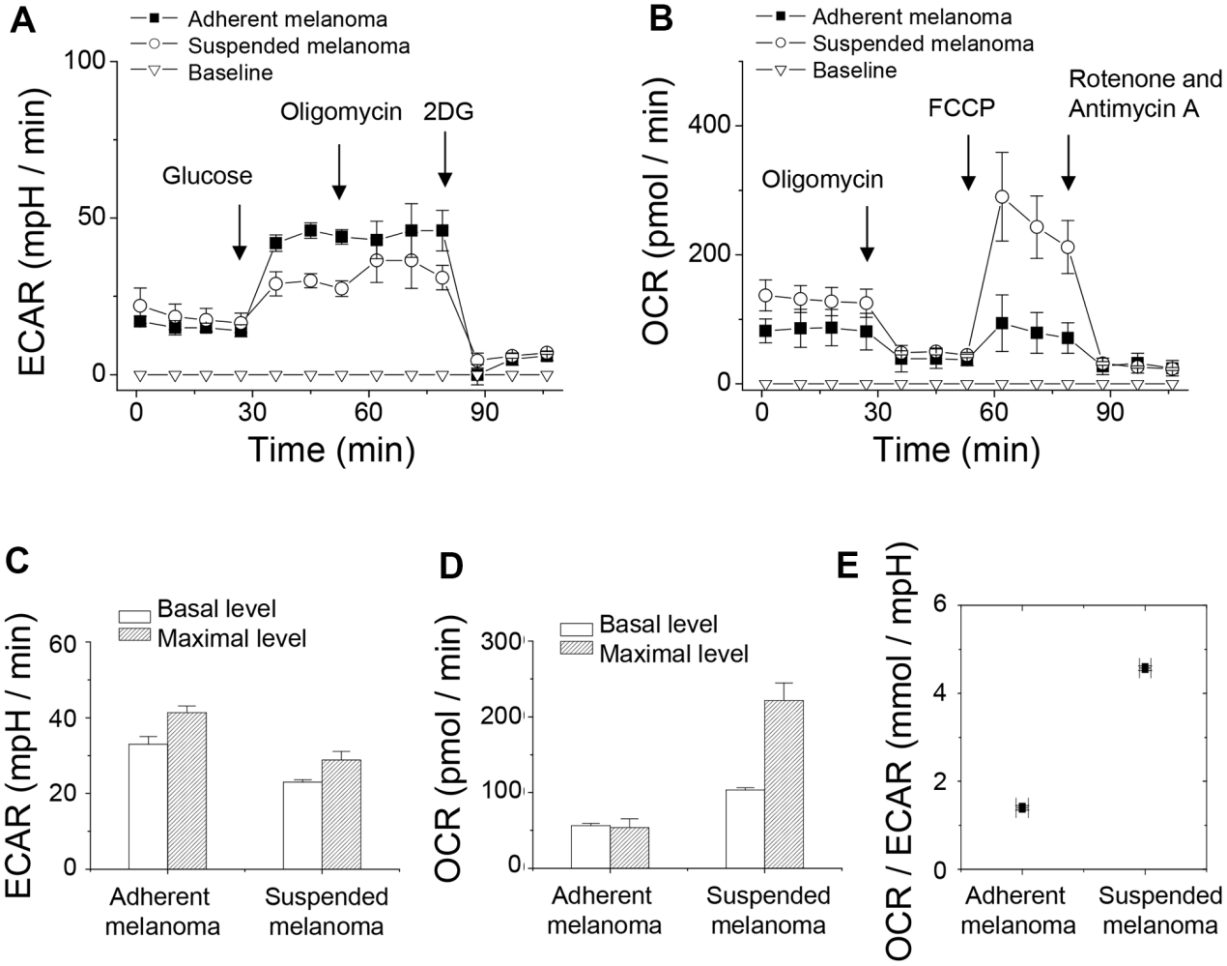
**Detachment stress decreased glycolytic capacity but increased respiratory capacity of melanoma A375 cells investigated by Seahorse XF24 analyzer.** (**A**) ECAR analysis showed higher rate of glycolysis in adherent melanoma cells. (**B**) OCR analysis showed higher oxygen consumption in suspended melanoma cells. (**C**) Decreased glycolytic capacity was observed in suspended melanoma cells, but ~80% glycolytic capacities were used in both adherent and suspended melanoma cells. (**D**) Increased respiratory capacity was seen in suspended melanoma cells. Adherent melanoma cells used full capacity in oxygen consumption, while suspended melanoma cells used only 46.7% of capacity. (**E**) Metabolism in suspended melanoma cells was highly dependent on mitochondrial respiration. Data were mean ±S.D. (n=3).

Extracellular acidification rate (ECAR) was the indicator of glycolysis. For evaluation of ECAR and glycolysis capacity, either adherent or suspended melanoma cells were initially incubated under a glucose-free medium. Extracellular acidification by lactate release was reported as pH change by the change in emission fluorescence of the pH-sensitive fluorophore. After sequential addition of glucose, oligomycin, and 2-deoxyglucose (2DG), the quantity of basal glycolysis, maximal glycolysis, and non-glycolytic acidification was determined. Oligomycin inhibits ATP synthase which reflected the amount of ATP production. 2DG is the glucose analogue that blocks glucose uptake. As seen in Figure 3A, suspended melanoma cells had a lower level of glycolysis, which was consistent with lower lactate secretion (Figure 1B). Upon addition of oligomycin, blockade of ATP synthase reflected maximal glycolysis, i.e. glycolytic capacity. This suggested that adherent melanoma cells were more glucose-dependent by higher glycolytic capacity than suspended melanoma cells (Figure 1A).

Oxygen consumption rate (OCR) was the indicator of mitochondrial respiration. For the evaluation of OCR, an O_2_-quenchable fluorophore was used to reflect O_2_ consumption. Sequentially additions of oligomycin, FCCP (*p*-trifluoromethoxyphenylhydrazone carbonyl cyanide), and rotenone were done to evaluate the change in OCR. Oligomycin inhibits ATP synthase which reflected the amount of ATP production. FCCP disrupted mitochondrial H^+^-gradient that showed maximal respiration. The combined action of rotenone and antimycin A inhibited electron transport chain that revealed the respiration level from non-mitochondrial sources. As seen in Figure 3B, the basal respiration rate of suspended melanoma cells was higher than that of adherent melanoma cells. The level of non-mitochondrial oxygen consumption was similar for both adherent and suspended melanoma cells (see the level after the addition of rotenone and antimycin A). These indicated that the overall ATP production from mitochondria source was higher in suspended melanoma cells. The maximal respiratory capacity for suspended melanoma cells was significantly larger than that for adherent cells (Figure 3B). This suggested that suspended melanoma cells harvested energy extensively from mitochondrial respiration.

Overly, the basal rate and maximal capacity of ECAR were lower but those of OCR were higher for suspended melanoma (Figure 3A, 3C). The difference between the basal rate and the maximum glycolytic capacity is defined as the glycolytic reserve. The glycolytic reserve for adherent and suspended melanoma cells was the same as 80% of full capacity. We explained the reduction in glycolytic capacity, but not the enzymatic conversion in glycolytic reactions under detachment stress. Reduced glucose uptake through reduced GLUT1 expression would account for this result.

Increased maximal respiratory capacity was found in suspended melanoma cells (Figure 3B, 3D). Approximate 4-folded increase in maximal respiratory capacity for melanoma cells under detachment stress. For adherent melanoma cells, the basal and maximal respiratory rates were equal, which indicated that saturated respiratory level and no spare respiratory capacity in adherent melanoma cells. However, the basal respiratory rate in suspended melanoma cells showed 46.7% saturated level (Figure 3D), which suggested enough spare capacity for energy production through mitochondrial respiration. It was reported that the increase in spare capacity implied the increased capacity of energy production in response to stress [18]. Mitochondrial spare respiratory capacity was the extra capacity to produce energy in response to increased stress and was associated with cellular survival. The preference of metabolic pathways could be estimated by the ratio of OCR to ECAR [19]. As seen in Figure 3E, the OCR/ECAR ratio in suspended melanoma cells was 3.3-folded higher than that in adherent melanoma cells. This implied the detachment stress enabled the metabolic switch of melanoma cells from Warburg phenotype into anti-Warburg phenotype.

### Detachment stress increased the superoxide production

**Figure 4:**
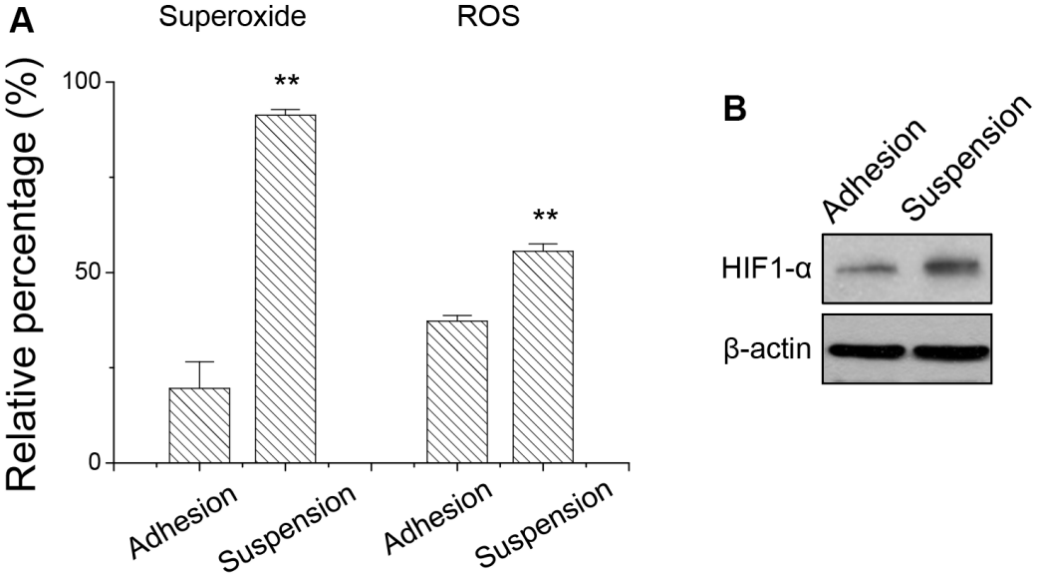
Detachment stress increased (**A**) the superoxide and ROS production, and (**B**) HIF-1α expression in melanoma A375 cells. Data were mean ±S.D. (n=3); **, *p* < 0.01.

Since suspended melanoma cells produced energy mostly through mitochondrial respiration, we examined whether the level of reactive oxygen species (ROS) in suspended melanoma cells was different from that in adherent melanoma cells. ROS include the superoxide anion (O_2_^−^), hydrogen peroxide (H_2_O_2_), and hydroxyl radicals (OH^·^), which are byproducts of mitochondrial respiration. ROS is associated with oxidative stress and leads to different pathology by damaging lipids, proteins, or DNA. As seen in Figure 4A, the relative levels of superoxide and ROS were significantly higher in melanoma cells under suspension. Especially, superoxide level was 4.7-folded higher for suspended melanoma cells compared with that of adherent melanoma cells. On the other side, the ROS level was 1.5-folded higher for suspended melanoma cells compared with that of adherent melanoma cells. This oxidative stress-induced HIF activation as seen by the elevated level of HIF-1α expression even under normoxia conditions (Figure 4B). The activated HIF pathway might be critical for downstream signaling and protein expression.

### Change in drug sensitivity of melanoma cells by cell detachment

**Figure 5:**
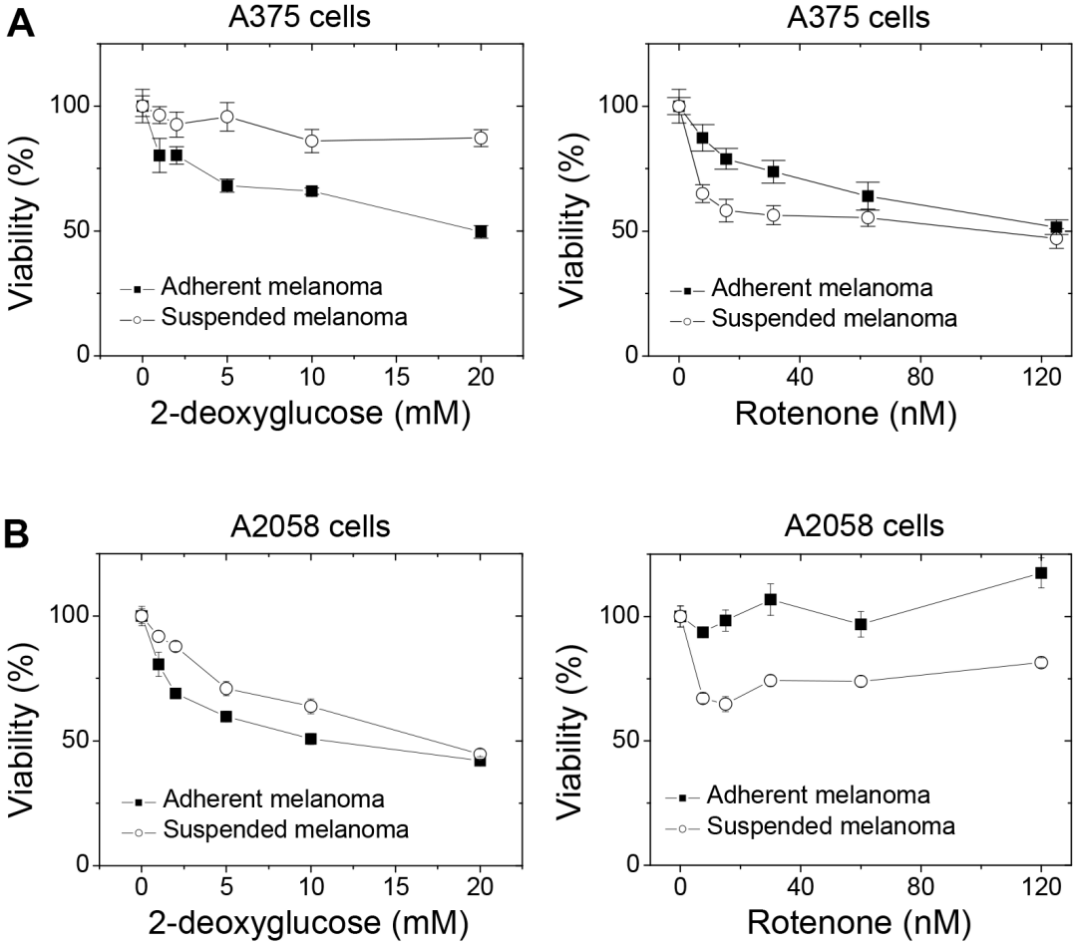
**Detachment stress altered the drug sensitivities toward the blockades of glycolysis and mitochondrial respiration in different melanoma cells.** Suspended melanoma cells were less sensitive to treatment of 2DG, but more sensitive to treatment of rotenone in (**A**) melanoma A375 cells and (**B**) melanoma A2058 cells. Data were mean ±S.D. (n=6).

We demonstrated that the proliferation of adherent melanoma cells was more sensitive to the reduced glucose concentration (Figure 1B) and was more glucose-dependent with higher glycolytic capacity. 2DG is the structural mimic of glucose that acts as a glycolysis inhibitor. As seen in Figure 5, increased 2DG concentrations resulted in decreased cell viability for adherent melanoma A375 and A2058 cells. However, increases in 2DG concentration had no significant effect on the viability of suspended melanoma cells. On the other side, the increase in maximal respiratory capacity and higher dependency on mitochondrial respiration in suspended melanoma cells would result in higher sensitivity toward blockade of electron transport chain. Rotenone is the inhibitor of complex I in electron transport chain. As seen in Figure 5, suspended melanoma cells were more sensitive to rotenone treatment in both A375 and A2058 cells. These results implied that detachment stress enabled metabolic switch from Warburg-like phenotype of higher glucose-dependency into anti-Warburg-like phenotype of higher mitochondrial respiration-dependency. In summary, different drug sensitivities on metabolism were presented in adherent and suspended melanoma cells.

## DISCUSSION

The transformed cancer cells presented one specific feature of survival under detachment stress, which was beneficial for their metastatic spread. This feature of anchorage-independent survival is also called anoikis resistance. Previously, we had demonstrated that melanoma cells could survive under anchorage independency [10]. Detachment stress also modified the surface of malignant melanoma cells and affected their interactions with microenvironments. Reduced syndecan-1 expression impaired the laminin-binding ability and their ability of cell invasiveness [10]. This downregulation of syndecan-1 was mediated by downregulation of aminopeptidase N, which was mediated by PKCδ activation and led to suppressed expression of integrin beta4 [12]. This generated different vascular phenotypes of melanoma tumors. In addition, detachment stress also increased the expression of IL-8 and CXCR1 through activation of ERK and JNK signaling, which led to increased tumor growth [11]. Detachment stress also affected the chemosensitivity of melanoma cells through upregulation of syndecan-2-mediated ERK/PI3K activation [13]. This evidence all suggested that detachment stress would be one of the prime factors to transform melanoma cells into different phenotypes.

Some literatures suggested the reprogramming of metabolism enabled cancer survival under detachment stress [20–22]. The interplay between acidic microenvironment, ROS production, and HIF activation was closely associated with metabolic pathways in cancer cells [22]. It was suggested that low extracellular pH promoted anoikis resistance through an increased N-cadherin expression with an enhanced migrative ability through the activation of epidermal growth factor receptor and Akt pathways. Our results showed the lactate secretion decreased as explained by the decreased expression of lactate transporter MCT4 (see Figure 2 and Table 3). However, our unpublished data showed N-cadherin expression level increased upon cell suspension, upon which the lower level of extracellular acidification would achieve by decreased lactate secretion. In addition, the released lactate was shown to be angiogenic factor or metastatic factor through the action of IL-8/VEGF or CD44, respectively [23]. Our previous observation [12] showed a lower vascular phenotype in suspended cell-derived tumors, which could be explained by the decreased lactate secretion. Another paper suggested that overexpression of pyruvate dehydrogenase kinase 4 (PDK4) would prolong cell survival under suspension, so that the phenotypes of decreased glucose oxidation and anoikis resistance were observed by cell detachment [24]. In our microarray results, the PDK4 levels were upregulated by comparing suspended cells to attached cells as seen in our microarray results. This strengthened our observation that cell detachment correlated with the scenario of glucose metabolism. In breast cancer cells, it was known that tumor cells generated ATP through fatty acid oxidation, but not glycolysis, under cell detachment [25]. It is possible that different neoplasia would acquire different mechanisms to overcome detachment stress.

It was reported that detachment stress causes mitochondria impairment and increased ROS production. The formation of cell clusters at the suspension stage induced the HIF-1α activation and promoted glycolysis. Perturbation of these metabolic adaptations resulted in ROS accumulation and cell death [26]. It’s also known the activation of transcription factor HIF-1 by lactate under hypoxia condition lead to tumor malignancy [27]. Our result (Figure 4B) also showed increased HIF-1α expression under detachment stress, however, the portion of glycolytic routes was decreased (Figure 3A, 3E). Production of superoxide might derive from either oxidation of NADPH by oxidase enzymes or from aerobic respiration in the mitochondria. Upon examining our previous microarray result (GSE42876), NOX4 expression was increased 1.74-folded upon cell detachment.

According to our previous studies and the current study, our conclusions suggested the detachment would actively promote the transformation of adherent melanoma cells into different phenotypes at cell invasiveness, tumor malignancy, chemosensitivity, as well as altered metabolism of anti-Warburg features. Although it’s hard to say the suspended cells were dormant (less invasive, less proliferative, and less glycolytic) or less “transformed”, we explained that detachment stress would enable the metastatic melanoma cells to survive under different environmental factors, such as immune system or chemotherapy.

## MATERIALS AND METHODS

### Cell culture and inhibitor treatments

Human melanoma A375, A2058, RPMI7951, and Hs695t cells were purchased from Bioresource Collection and Research Center (BCRC; Hsinchu, Taiwan) with authentication. The adherent culture was maintained using culture dish (Corning Incorporated Life Sciences, Tewksbury, MA, USA) and DMEM medium supplemented with 10% (v/v) fetal bovine serum (Biological Industries Ltd., Cromwell, CT, USA). Suspended melanoma cells were established and cultured by impaired attachment of adherent melanoma cells accordingly [28, 29]. In general, 4x10^5^ cells were cultured in sterile plastic dishes (Alpha-plus Inc., Taiwan) for 3 days before experiments. This generated the suspended melanoma cells.

Oligomycin (mitochondrial ATP synthase inhibitor), 2-deoxyglucose (2DG; glycolysis inhibitor), *p*-trifluoromethoxyphenylhydrazone carbonyl cyanide (FCCP; oxidative phosphorylation uncoupler), Rotenone and antimycin A (electron transport chain complex inhibitors) were all purchased from Sigma (Sigma-Aldrich China, Inc., Shanghai, PR China).

### Cell proliferation or cell viability assay

For cell proliferation under different glucose concentrations, 4x10^5^ cells were seeded at culture dish or sterile plastic dishes with indicated glucose concentrations. Cells were incubated in humidified CO_2_ incubator for 3 days. The cells were collected and the total cell numbers were counted by trypan blue methods.

For cell viability assay, it was assayed by Alamar blue method (Thermo Fisher Scientific Inc., Pittsburgh, PA, USA). In general, 1x10^4^ cells were seeded in each well of 96-well plate (Corning Incorporated Life Sciences, Tewksbury, MA, USA) pre-coated with or without polyHEMA. After overnight incubation, inhibitors were added and incubated for 1 day in culture incubators before the assays.

### Gene expression microarray and bioinformatics analysis

Total RNA was extracted using Trizol reagent (Invitrogen Taiwan Ltd., Taipei, Taiwan) and characterized to ensure good quality (OD_260_/OD_280_ >1.8) for array analysis. The following procedure for gene expression profiling using Illumina beadchips was similar to the published paper [30] with slight modification. The complementary RNAs were amplified and labeled by TotalPrep RNA amplification kit (Ambion Illumina Inc., USA). 1.5 ug of biotinylated cRNA were used in hybridization onto Human HT-12 v4 expression beadchip (Illumina Inc., USA) for 16 hr at hybridization chamber (58° C) according to manufacturer’s procedure. The beadchip was washed and the hybridized probes were detected by Cy3-streptavidin using Illumina BeadArray Reader (Illumina Inc., USA). Raw data of average probe signals were collected, background subtracted, and normalized by MBCB (model-based background correction for beadarray) method [31]. The array data were deposited in NCBI Gene Expression Omnibus database (http://www.ncbi.nlm.nih.gov/geo/) with the accession number of GSE198509.

### Lactate release assay and ROS detection assay

Quantification of lactate level was done using Lactate Colorimetric Assay Kit (Biovision, Inc., Milpitas, CA, USA) according to vendor’s protocol. Adherent or suspended cells (2x10^5^ cells) were incubated in the culture medium at pH7.0 or pH8.5 for 1 day without pH adjustment by CO_2_. The conditioned medium was collected and centrifuged for the following assay.

Detection of ROS levels in melanoma cells was done using ROS Detection Cell-Based Assay Kit (Cayman Chemical Co., Ann Arbor, MI, USA) according to vender’s protocol. Dihydroethidium is the redox-sensitive fluorescent probe. Its oxidation into 2-hydroxyethidium by superoxide or other ROS causes the increased fluorescence intensity at 600nM or 576nm. Antimycin A treatment and N-acetylcysteine treatment were positive and negative controls of ROS production, and used to calculate the relative percentage of ROS production.

### Polymerase chain reactions and statistical analysis

The levels of mRNA in cultured cells were analyzed by qPCR. The cDNAs were synthesized by MMLV HP reverse transcriptase (Epicentre Inc., Madison, WI, USA) using freshly prepared RNA as PCR template. Quantitative real-time PCR was performed using Fast Quant Green Master Mix with ROX (Protech Technology Enterprise Co., Ltd., NanKung, Taipei, Taiwan) in a StepOne Plus real-time PCR system (Thermo Fisher Scientific Inc., Pittsburgh, PA, USA). The 2^-ΔΔCT^ method was used to determine the relative gene expression using GAPDH as control. The *p*-value of < 0.05 or < 0.01 was statistically significant and was indicated in figures. The forward and reverse primers used were: MCT1 (SLC16A1), gtggctcagctccgtattgt and gagccgacctaaaagtggtg; MCT2 (SLC16A7), caacaccattccaagacagc and tggctgttatgtacgcagga; MCT4 (SLC16A3), cagttcgaggtgctcatgg and atgtagacgtgggtcgcat; GLUT1 (SLC2A1), ttgcaggcttctccaactggac and cagaaccaggagcacagtgaag; GAPDH, gagtcaacggatttggtcgt and gatctcgctcctggaagatg.

### Western blot and antibodies

To harvest cell lysate for western blot analysis, cells were washed and disrupted by lysis buffer (10 mM Tris-HCl, 5 mM EDTA, pH 8.0, 1% TritonX-100, and protease inhibitors) and kept on ice for 30 min. The lysate was then centrifuged at maximum speed using a desktop centrifuge at 4° C for 15 min. Protein concentrations were quantified by protein assay kit (Bio-Rad Laboratories Inc., Hercules, CA, USA).

Western blot was performed according to standard protocol. Briefly, the protein mixture was subjected to SDS-PAGE and transferred onto a PVDF membrane followed by blocking with 5% (w/v) skim-milk. The membrane was then incubated in primary antibodies (1:1000 in 5% skim-milk in TBST) overnight at 4° C, and HRP-conjugated secondary antibody (1:20000) for 1 hr at room temperature followed by enhanced chemiluminescent (Millipore Co., MA, USA) detection. The primary antibody against MCT1 (*SLC16A1*; ~37kDa), MCT2 (*SLC16A7*; ~35kDa), MCT4 (*SLC16A3*; ~40kDa), GLUT1 (*SLC2A1*; ~55kDa), HIF-1α (~110kDa), and β-actin were all purchased from GeneTex Inc., Hsinchu, Taiwan.

### Metabolism stress tests using Seahorse XF24 analyzer

The mitochondrial oxygen consumption rate, OCR, and the extracellular acidification rate, ECAR, were measured using a Seahorse Bioscience XF24 extracellular flux analyzer (Agilent Technologies, Inc., Santa Clara, CA, USA).

To prepare adherent melanoma cells, 5x10^4^ cells were seeded at Seahorse cell culture microplate overnight and reached 100% confluency on the next day. To prepare suspended melanoma cells, 5x10^4^ suspended cells were seeded on the experiment day at microplate precoated with poly-L-lysine. The sensor cartridge with the utility plate was assembled and incubated in the non-CO_2_ incubator at 37° C overnight before the assays.

For mitochondria stress test to measure OCR, culture medium was replaced and incubated with glucose-free/ serum-free/bicarbonate-free medium (D5030; Sigma-Aldrich China, Inc., Shanghai, PR China) for 1 hr before the assays. Mitochondria stress profiles were monitored after the successive injection of oligomycin (2 μM), FCCP (1 μM), and rotenone/antimycin A (1 μM each) to the indicated wells. Glycolysis stress profiles were monitored after the successive injection of glucose (10 mM), oligomycin (2 μM), and 2-deoxyglucose (2DG, 50 mM). OCR and ECAR results were analyzed using the Seahorse XF-24 software. Every point represents an average of three different wells. The cell numbers in each well were counted after the experiments, that were used to normalize the ECAR and OCR.
